# MicroRNA *miR-155* Activity in Mouse Choline Acetyltransferase-Positive Neurons Is Critical for the Rate of Early and Late Paraplegia After Transient Aortic Cross-Clamping

**DOI:** 10.3389/fnmol.2022.788301

**Published:** 2022-02-03

**Authors:** Hesham Kelani, Gerard Nuovo, Anna Bratasz, Jayanth Rajan, Alexander A. Efanov, Jean-Jacques Michaille, Hamdy Awad, Esmerina Tili

**Affiliations:** ^1^Department of Anesthesiology, Wexner Medical Center, The Ohio State University, Columbus, OH, United States; ^2^GNOME, Inc., Powell, OH, United States; ^3^Small Animal Imaging Center Shared Resource, Wexner Medical Center, The Ohio State University, Columbus, OH, United States; ^4^BioPerox-IL, Faculté des Sciences Gabriel, Université de Bourgogne-Franche Comté, Dijon, France; ^5^Department of Cancer Biology and Genetics, Wexner Medical Center, The Ohio State University, Columbus, OH, United States

**Keywords:** open repair (OR), aortic cross-clamping, spinal cord injury (SCI), *miR-155*, edema, motoneurons (MNs), endothelial cells (ECs), paraplegia

## Abstract

Aortic aneurism open repair surgery can cause spinal cord (SC) injury with 5–15% of patients developing paraparesis or paraplegia. Using a mouse model of transient aortic cross-clamping (ACC), we have previously found that the expression of proinflammatory microRNA *miR-155* increases in motoneurons (MNs) and endothelial cells (ECs) of ischemic SCs, and that global *miR-155* deletion decreases the percentage of paraplegia by 37.4% at 48-h post-ACC. Here, we investigated the cell-specific contribution of *miR-155* in choline acetyltransferase-positive (ChAT^+^) neurons (that include all MNs of the SC) and ECs to SC injury after ACC. Mice lacking *miR-155* in ChAT^+^ neurons (MN-*miR-155*-KO mice) developed 24.6% less paraplegia than control mice at 48-h post-ACC. In contrast, mice lacking *miR-155* in ECs (ECs-*miR-155*-KO mice) experienced the same percentage of paraplegia as control mice, despite presenting smaller central cord edema. Unexpectedly, mice overexpressing *miR-155* in ChAT^+^ neurons were less likely than control mice to develop early paraplegia during the first day post-ACC, however they reached the same percentage of paraplegia at 48-h. In addition, all mice overexpressing *miR-155* in ECs (ECs-*miR-155*-KI mice) were paraplegic at 48-h post-ACC. Altogether, our results suggest that *miR-155* activity in ChAT^+^ neurons protects the SC against ischemic injury during the first day post-ACC before becoming deleterious during the second day, which indicates that early and late paraplegias arise from different molecular malfunctions. These results point to the need to develop specific protective therapeutics aimed at inhibiting both the early and late deleterious events after open repair surgery of aortic aneurisms.

## Introduction

During thoracic-abdominal aortic aneurysm (TAAA) open chest repair (OR) surgery, the transient aortic occlusion due to aortic cross-clamping (ACC) creates a situation of acute hypoxia followed by rapid reperfusion in the spinal cord (SC), thus causing SC injury and paraplegia in about 5–10% of cases depending on the extent of aortic aneurism and duration of the procedure (Coselli et al., 2016, 2019; Moulakakis et al., 2018). The pathophysiology of ACC-induced SC injury, histopathological changes, and molecular mechanisms leading to paraplegia still remains elusive. Specifically, there is currently no preventive intervention given to patients, and cerebral spinal fluid (CSF) drainage and cooling are common practices used to protect TAAA patients from paraplegia (Khachatryan et al., 2021).

In our previously developed mouse model of OR (Awad et al., 2010), ACC-induced hindlimb paraplegia is usually delayed, with the majority of mice undergoing paraplegia approximately 44–48 h following ACC. The SC damage initiates in the gray matter interneurons, and is coupled with neuronal dropout, neuro-inflammation, vascular leakage, and central cord edema, that altogether culminate in paraplegia (Awad et al., 2010, 2018). Of note, the different OR animal models have in common to induce paraplegia within 48-h post-ACC, an observation also made in patients (Kakinohana et al., 2011; Coselli et al., 2016, 2019; Moulakakis et al., 2018; Awad et al., 2021a). We have previously shown that the expression of proinflammatory microRNA *miR-155* (a.k.a. *miR-155-5p*) increases sharply in the SC of wild-type (WT) mice that experience paraplegia after ACC, as compared with non-paraplegic mice (Awad et al., 2018). This upregulation was primarily in motoneurons (MNs) and endothelial cells (ECs) of the SC. Mice with global *miR-155* deletion developed 37.4% less paraplegia than WT mice, had less central cord edema and a better preservation of SC gray matter tissue after ACC (Awad et al., 2018). Hence, we hypothesized that *miR-155* activity in MNs, ECs or a combination of both contributes to paraplegia after ACC. *MiR-155* has been shown to have both, deleterious (Henry et al., 2019; Suofu et al., 2020) and protective (Harrison et al., 2017; Li et al., 2017) effects in animal models of brain traumatic injuries, suggesting that this microRNA plays specific roles in different phases of SC injury, by potentially targeting different sets of transcripts.

To dissociate the specific role of MNs and ECs within the neurovascular unit and the effects that *miR-155* has in both cell types, we developed knock-out (KO) mice that lack the expression of *miR-155* in choline acetyltransferase-positive (ChAT^+^) neurons, that include all MNs of the SC (MN-*miR-155*-KO), or in ECs (EC-*miR-155*-KO), as well as knock-in (KI) mice that overexpress *miR-155* specifically in ChAT^+^ neurons (MN-*miR-155*-KI) or ECs (EC-*miR-155*-KI). Using these four *miR-155* mouse genotypes and their littermates expressing *miR-155* normally as controls, we found that overexpressing or deleting *miR-155* in ChAT^+^ neurons respectively diminishes the percentage of early and late paraplegia post-ACC, while overexpressing *miR-155* in ECs increases the percentage of late paraplegia.

## Materials and Methods

### Animals

The Animal Care and Use Committee at the Ohio State University (OSU) approved all the experiments with animals. This investigation conforms to the Guide for the Care and Use of Laboratory Animals published by the NIH. C57Bl/6 mice were obtained from Jackson Laboratories. To prepare specific *miR-155* KO mice, transgenic *miR-155^fl/fl^* mice purchased from Jackson Lab were mated to either *ChAT-Cre*^+^ transgenic mice (Jackson Lab), which provides MN-*miR-155*-KO mice by driving the deletion of *miR-155* in ChAT^+^ neurons that include all motoneurons of the SC, or to *Tie2-Cre*^+^ transgenic mice (Jackson Lab), which gives EC-*miR-155*-KO mice by driving the deletion of *miR-155* in ECs. To overexpress *miR-155* in the desired cell types, we used transgenic *Rosa26^lox–stop–lox–miR–^*^155^ (*Rosa26*^LSL–miR–155^) mice that were prepared and donated by Dr. Croce (OSU) and will be described in detail in another report. These mice contain a *pri-miR-155* transgene inserted in the *Rosa26* locus, preceded by a *LoxP-Stop-LoxP* cassette that impairs its expression. The expression of the *pri-miR-155* transgene can be induced by expressing the Cre recombinase that removes *LoxP-Stop-LoxP* cassette. *Rosa26*^LSL–miR–155^ were thus mated to *ChAT-Cre*^+^ or *Tie2-Cre*^+^ transgenic mice to drive the expression of *miR-155* in ChAT^+^ neurons or ECs, respectively. Both male and female mice were used in our study.

### Aortic Cross-Clamping

Aortic cross-clamping was conducted the same day for 7.5 min as previously described in detail (Awad et al., 2010, 2018) on both control mice and mice with modified genotype to avoid environmental variations. The procedure is detailed in Supplementary Materials and Methods. Following ACC, given the rapidity of development of paraplegia associated with complete impairment of hindlimbs movements, mice were classified as “paraplegic” or “non-paraplegic” upon observation, without further functional testing.

### Magnetic Resonance Imaging Analysis

Magnetic resonance imagery (MRIs) were performed blindly at the Small Animal Imaging Core Shared Resource, OSU, using a Bruker BioSpec 94/30USR scanner operating at a field strength of 9.4 T (Bruker BioSpec, Germany). A 4-channels mouse brain phased array receiver-coil and 72 mm volume coil, as a transmitter were used. Anesthetized mice were placed on the holder in prone position, then the spine was gently flatted (when possible) and surface coil was placed over the dorsal side of mice covering upper lumbar and lower thoracic SC. Images were obtained using T2-weighted RARE sequences with the following parameters: TE/TR = 36/3,524 ms, Rare factor = 8, FOV = 15 × 17 mm, slice thickness = 0.5 mm, resolution = 58.6 × 66.4 μm, number of slices = 30, NA = 8. The last rib, and kidneys were used as landmark for conformation. The first slice was placed over the second disc below the last rib and 30 slices (15 mm) anterior have been acquired. The respiratory rate and rectal temperature were monitored through the experiment with a Small Animal Instrument unit (SAI, Inc., Stonybrook, NY, United States). For volumetric measurements, images were analyzed blindly to the genotype by manually tracing the SC and hyperintense region, which corresponds to edema, using in-house build software. Images and masks of edema has been visualized using open-source ITK-SNAP software^1^ (Yushkevich et al., 2006).

### Hematoxylin and Eosin Staining

Following euthanasia, mice were perfused, and their SC was collected and fixed in 4% formalin for 2 days, followed by embedding in paraffin. Cross-sections were prepared blindly then stained with hematoxylin and eosin (H&E) at the Mouse Pathology Laboratory at OSU. Sections were then analyzed blindly *vis-à-vis* both the genotype and the pathologic status (paraplegic or non-paraplegic).

### RNA Isolation and Quantitative Real-Time PCR

RNAs were extracted using TRIzol (Invitrogen, Carlsbad, CA, United States). The expression of *miR-155* was assessed using TaqMan^®^ 002571 assay. Values were normalized using *snoRNA135*, providing us with a relative level of expression in the different strains.

### Statistics

Quantitative real-time PCR tests and other quantitative analyses are presented as mean + SD and were compared using two-tailed Student’s *t*-tests. *p*-Values are given in the legends to figures. The percentages of fully paraplegic mice of different genotypes were compared using both a Chi-square test and a Fisher exact test.^2^

## Results

### Development of Mice That Either Overexpress or Lack *miR-155* in Choline Acetyltransferase-Positive Neurons or in Endothelial Cells

**FIGURE 1:**
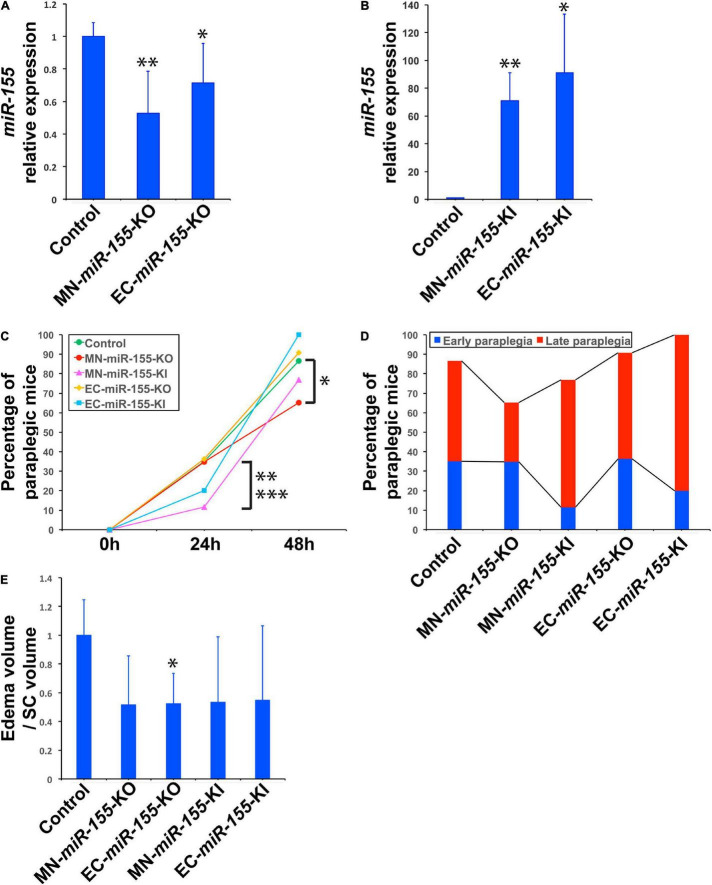
Deletion of *miR-155* in ChAT^+^ neurons protects mice against late paraplegia. **(A)** Real-time PCR analysis showing *miR-155* relative expression in the SC of MN-*miR-155*-KO and EC-*miR-155*-KO mice. *n* = 4 for each group. Values are given as mean ± SD. The mean value for control mice was arbitrarily set to 1. The symbols * and ^**^, significantly different from control. *p = 0.012; ^**^p = 0.055. **(B)** Real-time PCR analysis showing *miR-155* relative expression in the SC of MN-*miR-155*-KI and EC-*miR-155*-KI mice. *n* = 4 for each group. Values are given as mean ± SD. The mean value for control mice (identical to that in panel **A**) was arbitrarily set to 1. The symbols * and ^**^, significantly different from control. *p = 0.024; ^**^p = 0.0015. **(C)** Percentages of paraplegic mice at 24- and 48-h post-ACC as indicated for control (*n* = 37), MN-*miR-155*-KO (*n* = 23), EC-*miR-155*-KO (*n* = 11), MN-*miR-155*-KI (*n* = 26), and EC-*miR-155*-KI (*n* = 10) mice. *Significantly lower than control mice, *p* = 0.0519 (Chi-square) or *p* = 0.0627 (Fisher). ^**^Significantly lower than control mice, *p* = 0.0342 (Chi-square) or *p* = 0.0426 (Fisher). ^***^Significantly lower than MN-*miR-155*-KO mice, *p* = 0.0516 (Chi-square) or *p* = 0.0855 (Fisher). **(D)** Percentages of early and late paraplegia after ACC in control and genetically engineered mice. **(E)** Relative volumes of central cord edema in paraplegic control (*n* = 8), MN-*miR-155*-KO (*n* = 3), EC-*miR-155*-KO (*n* = 3), MN-*miR-155*-KI (*n* = 4), and EC-*miR-155*-KI (*n* = 5) mice at 48-h post-ACC. Edema volume, defined as the volume of increased T2 signal felt to represent central cord edema, was calculated by outlining areas of increased T2 signal. Relative values, calculated as ratios of edema volume/SC volume, are given as mean ± SD. The mean value for control mice was arbitrarily set to 1. *Significantly different from control, *p* = 0.031.

We have previously reported that *miR-155* is primarily upregulated in MNs and ECs of the SC of mice that undergo ACC-induced paraplegia, defined as the complete loss of capability to move their hindlimbs and tail, and that mice with global deletion for *miR-155* showed 37.4% less paraplegia than WT mice (Awad et al., 2018). To elucidate the specific contribution of these two cell types of the neurovascular unit to SC injury and paraplegia after ACC, and to determine how the functions of these cells is affected by the activity of *miR-155*, we developed two KO strains that specifically lack *miR-155* expression either in ChAT^+^ neurons (MN-*miR-155*-KO), or in ECs (EC-*miR-155*-KO), as well as two KI strains that overexpress *miR-155* specifically in ChAT^+^ neurons (MN-*miR-155*-KI) or ECs (EC-*miR-155*-KI). Of note, ChAT^+^ neurons include MNs of the SC plus a number of interneurons. Following genotyping of the offspring of each four crosses, extracts from the cervical SC of control mice and mice of the four above genotypes were analyzed for *miR-155* expression. The offspring of each cross that were either only *miR-155^fl/fl^*, *Rosa26*^LSL–miR–155^, *ChAT-Cre*^+^, or *Tie-Cre*^+^, i.e., that retained normal *miR-155* expression, were used as control mice. As expected, compared with control mice, *miR-155* basal expression was reduced in the SC of both MN-*miR-155*-KO and EC-*miR-155*-KO mice (Figure 1A). In contrast, *miR-155* expression was elevated in the SC of both MN-*miR-155*-KI and EC-*miR-155*-KI mice to levels comparable to those of a strong immune response (Figure 1B).

### The Deletion of *miR-155* in Choline Acetyltransferase-Positive Neurons Decreases the Percentage of Aortic Cross-Clamping -Induced Paraplegia at 48-h

The percentage of paraplegia after ACC surgery of mice of these four genotypes were then compared with that of control mice over a period of 48 h. The percentage of paraplegia at 24 h post-ACC was 11–36% depending on the strain (Figure 1C and Table 1). It subsequently increased sharply to reach 86.5% for control mice (*n* = 37), 76.9% for MN-*miR-155*-KI (*n* = 26), 90.9% for EC-*miR-155*-KO mice (*n* = 11), and 100% for EC-*miR-155*-KI mice (*n* = 10) at 48-h (Figure 1C and Table 1). In contrast, the percentage of paraplegia (65.2%) for MN-*miR-155*-KO mice (*n* = 23) was significantly lower (Chi-square: *p* = 0.0519; Fisher exact test: *p* = 0.0627) than that of control mice at 48-h post-ACC, indicating that *miR-155* activity in MNs and other ChAT^+^ neurons increases the risk of developing paraplegia. Of note, the 24.6% reduction of the percentage of paraplegia obtained by deleting *miR-155* only in ChAT^+^ neurons represents 65.8% of the reduction (37.4%) given by global *miR-155* deletion (Awad et al., 2018), suggesting that *miR-155* activity in other, non-ChAT^+^ cells of the SC may add to the deleterious effects of this microRNA after ACC. On the other hand, increasing *miR-155* activity in ECs proved detrimental, as all EC-*miR-155*-KI mice were paraplegic at 48-h, post-ACC. Altogether, these results indicate that *miR-155* activity in ChAT^+^ neurons is the main driver of SC injury at 48-h post-ACC.

**TABLE 1:** Dynamics of paraplegia development post-ACC for the different strains of mice.

Genotypes	Early paraplegia (0–24 h post-ACC)	Late paraplegia (24–48 h post-ACC)	Paraplegic mice at 48-h post-ACC	Non-paraplegic mice at 48-h post-ACC
Control (*n* = 37)	13 (35.1%)	19 (51.4%)	32 (86.5%)	5 (13.5%)
MN-*miR-155*-KO (*n* = 23)	8 (34.8%)	7 (30.4%)	15 (65.2%)	8 (34.8%)
MN-*miR-155*-KI (*n* = 26)	3 (11.5%)	17 (65.4%)	20 (76.9%)	6 (23.1%)
EC-*miR-155*-KO (*n* = 11)	4 (36.4%)	6 (54.5%)	10 (90.9%)	1 (9.1%)
EC-*miR-155*-KI (*n* = 10)	2 (20.0%)	8 (80.0%)	10 (100.0%)	0 (0.0%)

*The percentages of mice are given in brackets after the corresponding numbers of paraplegic or non-paraplegic mice.*

### Opposite Effects of *miR-155* Activity in Choline Acetyltransferase-Positive Neurons on Early and Late Paraplegia

Paraplegia after ACC occurs within 2 days in patients as well as in animal models, with a first wave occurring within hours post-ACC (early paraplegia) and a second wave taking place during the second day post-ACC (late paraplegia) (Kakinohana et al., 2011; Coselli et al., 2016, 2019; Moulakakis et al., 2018; Awad et al., 2021a). We therefore looked for *miR-155* effects during these two waves. Unexpectedly, *miR-155* activity protected mice against early paraplegia, for increasing *miR-155* activity in ChAT^+^ neurons (MN-*miR-155*-KI mice) reduced the percentage of paraplegia at 24-h post-ACC by three times as compared with either control mice (Chi-square: *p* = 0.0342; Fisher exact test: *p* = 0.0426) or MN-*miR-155*-KO mice (Chi-square: *p* = 0.0516; Fisher exact test: *p* = 0.0855) (Figures 1C,D and Table 1). There also was a possible tendency to reduced early paraplegia of EC-*miR-155*-KI mice, although it did not reach statistical significance. On the other hand, neither *miR-155* deletion in ChAT^+^ neurons nor in ECs had any measurable effect against early paraplegia (Figures 1C,D and Table 1). In contrast, the percentage of late paraplegia was lower in MN-*miR-155*-KO mice but higher in MN-*miR-155*-KI mice, indicating that *miR-155* deleterious effects in ChAT^+^ neurons primarily occur during the second day post-ACC in relation with *miR-155* intraspinal proinflammatory effects (Figure 1D and Table 1). In addition, overexpressing *miR-155* in ECs (EC-*miR-155*-KI mice) proved highly deleterious during the second day post-ACC, with the remaining 80% of mice developing late paraplegia.

Altogether, these results suggest that: (i) *miR-155* activity in ChAT+ neurons is protective during the first 24 h post-ACC, when the formation of reactive oxygen species causes mitochondrial damage, leading to excitotoxicity followed by cytogenic edema (Juurlink and Paterson, 1998); and (ii) *miR-155* activity in both ChAT^+^ neurons and ECs increases SC injury during the second day post-ACC, a period marked by the rapid expansion of the central cord (gray matter) edema in relation with increased inflammation and damage to the blood-SC barrier (see hereafter).

### The Deletion of *miR-155* in Choline Acetyltransferase-Positive Neurons and Endothelial Cells Is Associated With a Modified Pattern of Central Cord Edema

**FIGURE 2:**
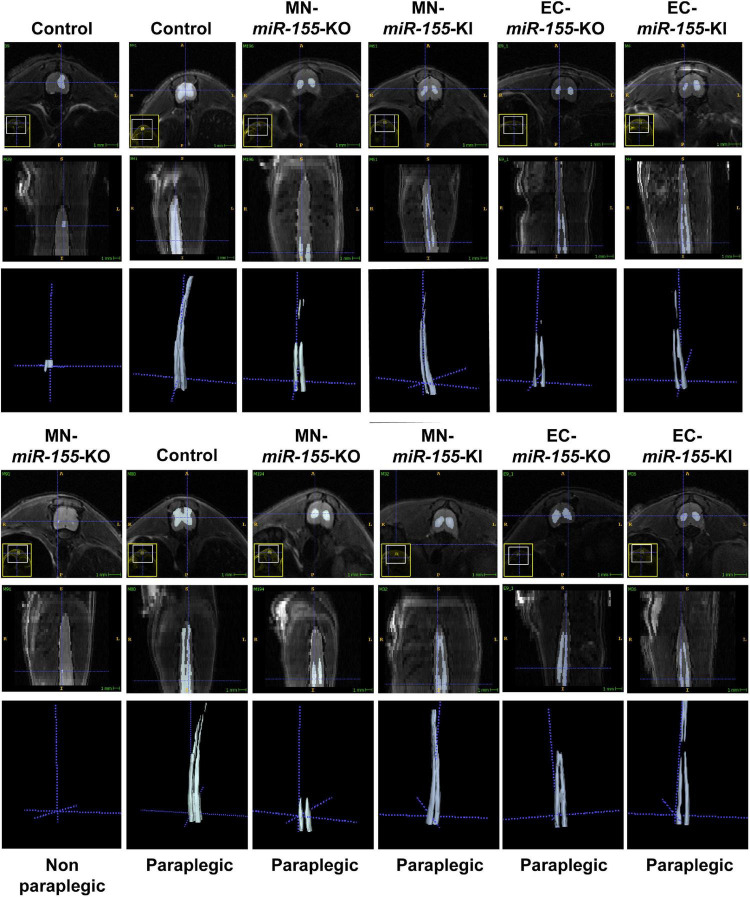
Deletion of *miR-155* in EC modifies the pattern and/or the volume of central cord edema 48-h post-ACC. Representative axial (top rows) and reconstructed coronal (bottom rows) T2-weighted images of thoracic and lumbar SCs from control, MN-*miR-155*-KO, MN-*miR-155*-KI, EC-*miR-155*-KO, and EC-*miR-155*-KI mice as indicated. Mice were either non-paraplegic (first left column) or paraplegic (second to sixth columns).

We have previously reported that ACC leads to the development of central cord edema associated with increased *miR-155* expression at 48-h post-ACC (Awad et al., 2018). We have also reported that the development of central cord edema is somewhat delayed in *miR-155* global KO mice, based on the fact that paraplegic *miR-155* global KO mice showed a reduced volume of edema at 48-h post-ACC as compared with WT mice. As we previously reported (Awad et al., 2018), non-paraplegic control or MN-*miR-155*-KO mice showed minimal edema at 48-h post-ACC, while paraplegic control mice in contrast showed very large edema including both ventral and dorsal horns of the SC and extending from the lumbar toward the cervical region of the SC (Figure 2 and Supplementary Figure 1). Strikingly, the edema in two out of three imaged paraplegic MN-*miR-155*-KO mice was restricted to the lumbar region of the SC (Figure 2). The edema in paraplegic EC-*miR-155*-KO also did not extend as far toward the cervical region as in control mice. In contrast, the edema in paraplegic EC-*miR-155*-KI and MN-*miR-155*-KI mice extended anteriorly like in control mice (Figures 1C, 2 and Supplementary Figure 1).

**FIGURE 3:**
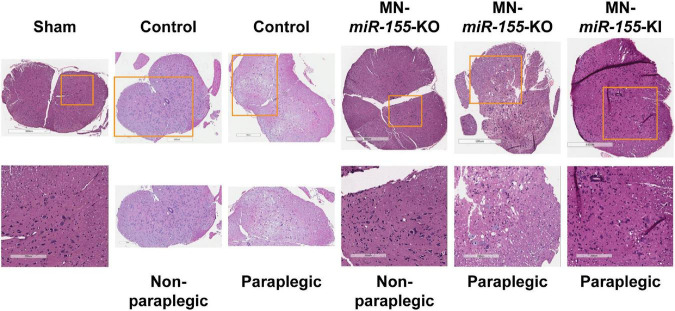
The level of expression of *miR-155* in ChAT^+^ neurons correlates with the extent of histological damage to the SC gray matter. H&E staining of representative cross-sections of the lumbar SC from sham (surgery procedure without ACC) as well as from control, MN-*miR-155*-KO and MN-*miR-155*-KI ACC-mice, either paraplegic or non-paraplegic, as indicated. Bottom row pictures show enlargements from the respective top row pictures.

Volumetric measures showed a tendency toward reduced edema volume in paraplegic mice of all the genetically engineered strains, however this volume reduction reached significance in EC-*miR-155*-KO mice only, possibly due the large variation in edema volume at the time of paraplegia that translated into large standard deviations (Figure 1E). Remarkably, while MN-*miR-155*-KO mice had a reduced percentage of paraplegia as compared with control mice at 48-h post-ACC (Figure 1C) and developed a central cord edema restricted to the lumbar part of the SC (Figure 2), the average volume of their edema was not significantly smaller than that of control mice or of the other genetically engineered mice (Figure 1E). This result suggests that *miR-155* deletion in ChAT^+^ neurons reduces the anterior spreading of the inflammation that develops during the second day after ACC rather than the intensity of the inflammatory response. On the other hand, EC-*miR-155*-KO, whose percentage of paraplegia post-ACC was similar to that of control mice at 48-h (Figure 1C), showed an average edema volume approximately 50% smaller than that of control mice (Figures 1E, 2 and Table 1). This result is not surprising, for ECs are responsible of the maintenance of the stability of the blood-SC barrier. Of note, while all EC-*miR-155*-KI mice were paraplegic at 48-h post-ACC (Figure 1C), their average edema volumes was not significantly different from that of control mice (Figure 1E). This was possibly due to edema fluid leaking out of the SC, given that EC-*miR-155*-KI mice already showed hemosiderin deposition—a sign of vascular leakage—in different tissue before ACC (not shown). The above results indicate that: (i) there is no direct correlation between either the pattern of edema or the average volume of edema with the percentage of paraplegia at 48-h post-ACC; and (ii) the development and anterior spreading of central cord edema is more likely caused by the inflammation that develops during the second day post-ACC, and whose level is primarily determined by *miR-155* expression in ChAT^+^ neurons. In agreement with the above conclusion, the lumbar SC of a paraplegic MN-*miR-155*-KO mouse at the time of paraplegia presented with significantly vacuolated gray matter—that contains interneurons and the cell body of MNs—with overall white matter sparing, similar to the SC of paraplegic control mice (Figure 3). In contrast, the damage to MNs was more limited in the gray matter of a MN-*miR-155*-KI mouse that had a percentage of paraplegia similar to that of control mice.

## Discussion

The enlargement of aneurisms of thoracic-abdominal aorta can lead to rupture and death. Currently, two types of TAAA repair surgery are used: OR surgery, that requires ACC to replace the weak and bulging section of the aorta by a graft, and endovascular repair, that consists of stenting the aorta through the femoral artery. We recently developed two dog models that allowed us to compare the pathophysiology of paraplegia after these two surgical procedures, and found that, in contrast to open repair that causes massive gray matter damage and neuronal death as a result of transient, severe ischemia followed by rapid reperfusion, endovascular repair primarily induces white matter damage associated with limited neuronal death as a consequence of extended hypoperfusion (Awad et al., 2021b). As there is presently no preventive pharmaceutical treatment against complications for either procedure, and given the frequency of aortic aneurisms (6–10 per every 100,000 people, with 9,923 deaths recorded in the United States in 2018), it is critical to understand the molecular dysfunctions that lead to similar percentages of paraplegia after these two surgical procedures (Rocha et al., 2020). While the advent of paraplegia after aortic stenting is most often delayed for weeks or even months, OR is especially challenging due to the fast extent of gray matter damage and the very short window of time (2 days) available for preventive intervention before the advent of permanent paraplegia, in human as well in animal models (Kakinohana et al., 2011; Coselli et al., 2016, 2019; Moulakakis et al., 2018; Awad et al., 2021a_)_. Using our mouse model of transient ACC, we had previously shown that *miR-155* global deletion reduces the risk of developing paraplegia by 37.4% at 48-h (Awad et al., 2018). This result however raised two questions: (i) In which cells of the neurovascular unit are *miR-155* initial, critical deleterious molecular effects are taking place? and (ii) When are deleterious molecular changes leading to paraplegia taking place following ACC?

In the present study, we developed four new types of genetically engineered mice to analyze the effects of *miR-155* activity in ChAT^+^ neurons and in ECs, that we both previously found to be involved in deleterious events leading to paraplegia (Awad et al., 2018). Our main findings are that: (i) deletion of *miR-155* in ChAT^+^ neurons, that include the MNs of the SC, reduced the anterior spreading of central cord edema and decreased the percentage of paraplegia at 48-h by 24.6%, primarily by reducing the percentage of late paraplegia (second day post-ACC); (ii) overexpressing *miR-155* in ChAT^+^ neurons, in contrast, decreased the percentage of early paraplegia (first day post-ACC) without significantly affecting the percentage or paraplegia at 48-h post-ACC; (iii) *miR-155* deletion in ECs remained without measurable effects on the percentage of paraplegia, however it reduced by 50% the volume of central cord edema as well as its anterior spreading; and (iv) overexpressing *miR-155* in ECs led to 100% of mice developing paraplegia.

One critical finding in this study is that *miR-155* activity in ChAT^+^ neurons was instrumental in molecular malfunctions causing late paraplegia. This is a groundbreaking result, considering the broad literature suggesting that *miR-155* exerts its pro-inflammatory, deleterious effects primarily in cells of the myeloid lineage and that glia-related neuro-inflammation is the main cause of most neuropathologies. Accordingly, a *miR-15*5 antisense inhibitory RNA reduced brain injury and improved recovery in a mouse model of stroke by distal middle cerebral artery occlusion (Caballero-Garrido et al., 2015). This finding fits well with our results, given that the injection of inhibitory RNAs was conducted for 3 days starting only at 48-h post-occlusion, meaning that the inhibitory RNAs were not interfering with the activity of *miR-155* during the early phase of stroke. Of note, such a delayed intervention would not be possible after ACC, for at 48-h post-ACC mice or patients with severe SC injury are already irreversibly paraplegic.

A second key finding is that *miR-155* activity in ChAT^+^ neurons in contrast reduced the initial extent of SC damage caused by ischemia/reperfusion. In agreement with this result, *miR-155* had a neuroprotective effect after mouse traumatic brain injury, and mice with global *miR-155* deletion showed increased neuronal degeneration, in particular by decreasing the expression of genes encoding Interferon α2, α4, α5, and β1 (Harrison et al., 2017). In addition, pretreating rats with the flavonoid formonetin before brain traumatic injury increased *miR-155* and *Heme oxygenase-1* expression (Li et al., 2017), the latter through targeting of *Bach1*, a repressor of several genes implicated in protection against oxidative stress. *MiR-155* also increased the survival of cardiomyocyte progenitor cells caused by necrotic cell death during a 16-h oxidative-stress stimulation (Liu et al., 2011). These two findings point to the necessity of distinguishing *miR-155* intrinsic effects on neuron homeostasis and survival at both the early and the late stages after OR. Overall, the findings suggest that *miR-155* targets different sets of transcripts during the early and the late hours after ACC.

In the OR, a short period of ischemia caused by ACC is followed by a wave of reperfusion that induces the formation of reactive oxygen species and mitochondrial damage, and causes excitotoxicity followed by cytogenic edema (Juurlink and Paterson, 1998). Our results suggest that *miR-155* may limit SC injury at this early stage. Later on, an exacerbated inflammatory response leads to the development of vasogenic edema, in particular due to *miR-155* damaging the blood-SC barrier (Lopez-Ramirez et al., 2014). Indeed, vasogenic edema has been shown to be associated with damage to the blood-SC barrier, in particular, due to *miR-155* inhibitory effects on transcripts encoding tight junction protein 1 (TJP1/ZO-1) (Caballero-Garrido et al., 2015). In addition, we have previously associated delayed paraplegia at 48-h post-ACC with *miR-155* targeting transcripts encoding major facilitator superfamily domain containing 2A (Mfsd2a) (Awad et al., 2018), a transporter implicated in the maintenance of the integrity of the blood-brain barrier (Ben-Zvi et al., 2014) that also delivers docosahexaenoic acid (DHA) to the brain (Nguyen et al., 2014). DHA in particular is a precursor of anti-inflammatory neuroprotectin-D1, that protects the brain and retina against cell injury-induced oxidative stress (Asatryan and Bazan, 2017). Thus, it is not surprising that *miR-155* deletion in ECs reduced the edema at 48-h post-ACC. In this respect, it will be interesting to understand how *miR-155* expression in ChAT^+^ neurons and ECs can affect the pattern of central cord edema. Finally, the different effects of *miR-155* on neuronal homeostasis at early and late stages are likely to result at least in part from ACC-induced modifications of the neuronal transcriptome and thus of changes in available *miR-155* target transcripts.

A third critical finding in our study is the discovery that the early and late paraplegias induced by ACC are caused by the malfunction of different molecular pathways, and thus that the progression of paraplegia is not linear. Supporting our findings, it has been shown that ACC for 5 min gives rise to neuronal loss and late paraplegia during the second day post-ACC, while ACC for 9 min causes early paraplegia associated with neuronal loss taking place between 8- and 24-h post-ACC (Kakinohana et al., 2011). It was further shown that Caspase 3 activation in neurons was taking place almost exclusively in mice submitted to 5-min of ACC. Strikingly, deleting the gene encoding Caspase 3 protected mice against late paraplegia after 5-min ACC, but not against early paraplegia after 9-min of ACC, indicating that the mechanisms responsible for early and late neuronal death are different (Kakinohana et al., 2011).

On the other hand, *miR-155* is implicated in the production of pro-inflammatory cytokines and chemokines (Tili et al., 2007, 2013). Cytokines and chemokines, produced by microglia, neurons, astrocytes, and ECs, can attract leukocytes such as neutrophils, monocytes, and lymphocytes (Kim et al., 2016). Once activated, these cells participate to further increasing the levels of inflammation and inducing neuronal death (Cartier et al., 2005; Ramesh et al., 2013). Of note, proinflammatory cytokines IL-1β and TNF as well as chemokines KC/IL-8/CXCL8 and MIP1α present a biphasic response to 4-min ACC, with a peak at 6-h and a peak at 36-h post-ACC. In contrast, chemokines MCP1/CCL2, IL-6, and RANTES only peaked at 36-h post-ACC (Smith et al., 2012). In the light of our results, we can speculate that the cytokines and chemokines produced at 6- and 36-h post-ACC play a role in both early and late paraplegia as well as in edema formation, while those produced only at 36-h rather participate to exacerbating inflammation and edema extension and concur to late paraplegia.

As a further complication, *miR-155* has dose-dependent effects, and targets different sets of transcripts when expressed at different doses (Tili et al., 2015; Narayan et al., 2017; Michaille et al., 2019). *MiR-155* expression at 48-h post-ACC is much higher in the SC of paraplegic mice than in the SC of non-paraplegic mice, and is particularly elevated in the MNs of the ventral horns (Awad et al., 2018). It is thus conceivable that both increasing *miR-155* levels at the early stage and impairing its expression or blocking its activity at the later stage post-ACC would be beneficial. It will thus be critical to use massive sequencing along with proteome, Metabolome, and bioinformatics analyses to identify the key miR-155 targets that are responsible the protective and deleterious effects of this microRNA during day-1 and day-2 post-ACC, respectively.

## Conclusion

In summary, our present findings, in light of previously published reports, define two windows of time during which different molecular deleterious events cause SC injury and paraplegia post-ACC, thus calling for the development of timely targeted preventive therapeutics specifically tailored to prevent neuronal damage during both the early and late phases following ischemia/reperfusion.

## Data Availability Statement

The original contributions presented in the study are included in the article/Supplementary Material, further inquiries can be directed to the corresponding authors.

## Ethics Statement

The animal study was reviewed and approved by the Animal Care and Use Committee at The Ohio State University.

## Author Contributions

ET, HA, and J-JM conceived and designed the experiments. HK, GN, AB, JR, and AE performed the experiments. ET, HA, HK, and J-JM analyzed the data and wrote the manuscript. All authors contributed to the article and approved the submitted version.

## Conflict of Interest

GN is employed by GNOME, Inc., Powell, OH, United States. The remaining authors declare that the research was conducted in the absence of any commercial or financial relationships that could be construed as a potential conflict of interest.

## Publisher’s Note

All claims expressed in this article are solely those of the authors and do not necessarily represent those of their affiliated organizations, or those of the publisher, the editors and the reviewers. Any product that may be evaluated in this article, or claim that may be made by its manufacturer, is not guaranteed or endorsed by the publisher.

## Footnotes

1
http://www.itksnap.org

2
https://www.socscistatistics.com
