# Supplementary material for: MicroRNA miR-155 Activity in Mouse Choline Acetyltransferase-Positive Neurons Is Critical for the Rate of Early and Late Paraplegia After Transient Aortic Cross-Clamping
Source: Front Mol Neurosci. 2022 Feb 3;15:788301. doi: 10.3389/fnmol.2022.788301 (PMC8850917; doi:10.3389/fnmol.2022.788301)
Supplement: Supplementary file 1 [file Data_Sheet_1.PDF]

Supplementary Materials and Methods

Mice were anesthetized with Ketamine/Xylazine and maintained under anesthesia with 1.5-2.5% isoflurane in oxygen enriched air for the duration of the surgical procedure, which is generally 60-90 minutes in length. The neck was incised longitudinally, an endotracheal tube was inserted and the animal was kept on a ventilator. With the animals in the left lateral position, a left thoracotomy was performed in the second intercostal space, care being taken to avoid incising the left superior vena cava. The aortic arch was gently isolated between the left common carotid artery and the left subclavian artery (LSA), avoiding the vagus nerve and the left recurrent laryngeal nerve. Then, under direct vision, one clip was placed on the aortic arch between the left common carotid artery and the LSA, and then another clip was placed on the origin of the LSA (within 15 seconds). The vascular occlusion was verified and maintained for 7.5 minutes. Following occlusion, the clips were removed, and the chest was closed with absorbable PDS in layers, first the thoracic cage, followed by the muscular layer, and then finally the skin. The suture size for the rib cage was 6.0 Nylon and 5.0 Nylon for the skin. After 15 minutes of warming up, the neck incision was closed before mice recovered from anesthesia, and the endotracheal tube was removed once mice were weaned from the ventilator and breathing without assistance. Mice were then placed in a cage kept at ~31-34ºC.
