# Supplementary material for: MicroRNA miR-155 Activity in Mouse Choline Acetyltransferase-Positive Neurons Is Critical for the Rate of Early and Late Paraplegia After Transient Aortic Cross-Clamping
Source: Front Mol Neurosci. 2022 Feb 3;15:788301. doi: 10.3389/fnmol.2022.788301 (PMC8850917; doi:10.3389/fnmol.2022.788301)
Supplement: Supplementary file 2 [file Presentation_1.PPTX]

## Slide 1
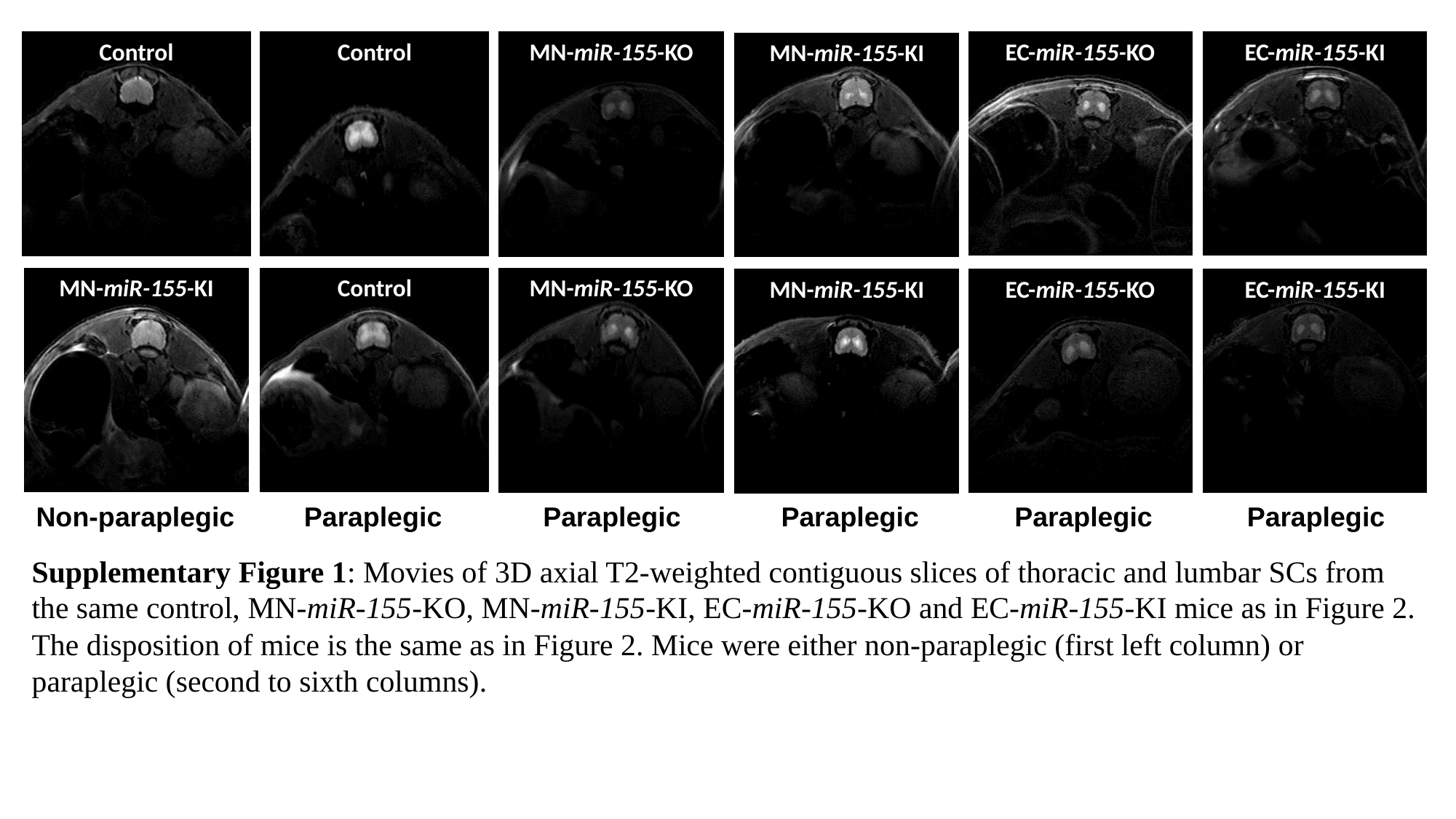

Control
MN-miR-155-KI
Control
Control
MN-miR-155-KO
MN-miR-155-KO
EC-miR-155-KO
EC-miR-155-KO
EC-miR-155-KI
MN-miR-155-KI
MN-miR-155-KI
EC-miR-155-KI
Non-paraplegic
Paraplegic
Paraplegic
Paraplegic
Paraplegic
Paraplegic
Supplementary Figure 1: Movies of 3D axial T2-weighted contiguous slices of thoracic and lumbar SCs from the same control, MN-miR-155-KO, MN-miR-155-KI, EC-miR-155-KO and EC-miR-155-KI mice as in Figure 2. The disposition of mice is the same as in Figure 2. Mice were either non-paraplegic (first left column) or paraplegic (second to sixth columns).
